# P356: Serious complications of childbirth in the university hospital of Yaoundé: retrospective study over 10 years

**DOI:** 10.1186/2047-2994-2-S1-P356

**Published:** 2013-06-20

**Authors:** W Takang, F Fouakeng

**Affiliations:** 1 CHU Yaoundé, Yaoundé, Cameroon

## Introduction

Severe complications of childbirth contribute significantly to the increase in overall rates of maternal and neonatal mortality and infant are targets of the MDGs.

## Objectives

To determine the frequency and factors involved in serious complications of childbirth at the university hospital pf Yaounde; to make recommendations regarding quality of care for childbirth in health facilities in the country.

## Methods

A descriptive retrospective chart review study between 1 January 2000 to 31 December 2009 at the Hospital of Yaoundé of all parturients who presented: a intrapartum uterine rupture before or after admission to the maternity ward (room delivery), maternal death and fetal intra or postpartum and early neonatal or perinatal death.

## Results

Among 16,733 recorded births, the following was noted: 40 cases of uterine rupture in parturients came from a private health center or district hospital (70% of cases) with use of oxytocin in 40% of cases. The main contributing factors were a scarred uterus, multiparity, a large fetus weighing> 3500 g and use oxytocin.

In 70% of cases of uterine rupture was corporeal, surgery performed after hysterosalpingography in 65% of cases. Perinatal mortality associated with uterine rupture was 94.7% and maternal mortality of 7.5%.

## Conclusion

Thus, we must multiply the observed rates of uterine rupture, perinatal and maternal mortality by a factor of at least 3 for the same reasons that the results of this referral hospital in the capital of the country, are representative of the situation of serious complications of childbirth in the country.

## Disclosure of interest

None declared.

